# Deep learning for forensic age estimation using orthopantomograms in children, adolescents, and young adults

**DOI:** 10.1007/s00330-025-11373-y

**Published:** 2025-01-25

**Authors:** Rahel Mara Koch, Hans-Joachim Mentzel, Andreas Heinrich

**Affiliations:** 1 Department of Radiology, Jena University Hospital—Friedrich Schiller University, Am Klinikum 1, 07747 Jena, Germany; 2 Section of Pediatric Radiology, Department of Radiology, Jena University Hospital—Friedrich Schiller University, Am Klinikum 1, 07747 Jena, Germany

**Keywords:** Age determination by teeth, Deep learning, Forensic medicine, Neural network models, Panoramic radiography

## Abstract

**Objectives:**

Forensic age estimation from orthopantomograms (OPGs) can be performed more quickly and accurately using convolutional neural networks (CNNs), making them an ideal extension to standard forensic age estimation methods. This study evaluates improvements in forensic age prediction for children, adolescents, and young adults by training a custom CNN from a previous study, using a larger, diverse dataset with a focus on dental growth features.

**Methods:**

21,814 OPGs from 13,766 individuals aged 1 to under 25 years were utilized. The custom CNN underwent 1000 epochs of training and validation using 16,000 and 4000 OPGs, respectively. The best model was chosen by the least mean absolute error (MAE) and evaluated with an additional test dataset of 1814 independent OPGs. Furthermore, the CNN was applied to OPGs from 15 available forensic age estimations conducted by experts certified by the Study Group on Forensic Age Diagnostics (AGFAD), and the results were compared.

**Results:**

A MAE of 0.93 ± 0.81 years and a mean-signed error (MSE) of −0.06 ± 1.23 years were achieved in the test dataset. 63% of predictions were accurate within 1 year, and 95% within 2.5 years. Results of the CNN were comparable to those obtained by experts, effectively highlighting discrepancies in the reported ages of individuals.

**Conclusion:**

Using a large and diverse dataset along with custom deep learning techniques, forensic age estimation can be significantly improved, often providing predictions accurate to within 1 year. This approach offers a reliable, robust, and objective complement to standard forensic age estimation methods.

**Key Points:**

***Question*** *The potential of custom convolutional neural networks for forensic age estimation, along with a large, diverse dataset, warrants further investigation, offering valuable support to experts.*

***Findings*** *For 1814 test-orthopantomograms, 63% of predictions were accurate within 1 year and 95% within 2.5 years, similar to expert estimates in 15 forensic cases.*

***Clinical relevance*** *Many individuals’ fates depend on accurate age estimation. Forensic age estimation can benefit from applying CNN-based methods to further enhance reliability and accuracy.*

## Introduction

Forensic age estimation is vital in forensic science, law enforcement, and virtual autopsy, relying on maturity indicators like tooth and bone development [1–3], which are consistent across individuals. The Study Group on Forensic Age Diagnostics of the German Society of Legal Medicine (AGFAD) recommends a three-step approach for legal cases involving adolescents and young adults: medical history and physical examination, followed by radiography of the left hand/wrist and orthopantomography (OPG) of the jaw [2, 4, 5]. Additionally, computed tomography (CT) examinations of the clavicles are performed once hand/wrist development is complete. OPGs are particularly valuable for age estimation, as they provide detailed insights into dental factors such as development, mineralization, root growth, and eruption sequence [2, 3].

A novel method for estimating age using OPGs involves convolutional neural networks (CNNs) [6–17]. While many approaches yield good results, they often focus on specific teeth [6, 9, 10, 12, 15, 17], limiting their applicability. Most methods classify [6, 7, 9, 10, 14] rather than accurately estimate age and rely on small datasets [6–9, 12–15, 17] and transfer learning models [7, 9–11, 14, 16, 17]. A prior study [18] developed a custom CNN that estimates age from 2 to 89 years, surpassing transfer learning models in accuracy. A wide variety of OPGs were used, including digitized OPGs from X-ray films, which typically have poorer image quality, affecting CNN accuracy [8]. The goal was to create a robust model that provides consistent predictions across all ages, including for postmortem OPGs, even with variations in input quality, accepting a slight reduction in accuracy to facilitate rapid person identification through matching with large databases [18, 19]. However, separating the age estimation methods into (1) children, adolescents, and young adults, and (2) adults could improve the results [9, 18], as the CNN would learn specific features related to only dental growth (mineralization, root growth, eruption) rather than general aging processes (attrition, root resorption, tooth loss, etc.).

This study aims to enhance age estimation in children, adolescents, and young adults by training the custom CNN with an expanded dataset of 21,814 OPGs (compared to 11,638 OPGs in the previous study for the same age range), which is expected to improve accuracy [16, 18]. The CNN focuses on dental growth features by limiting the age to 25 years and excluding digitized OPGs. Additionally, the CNN results of OPGs from 15 real forensic age estimations were compared with those conducted by experts certified by AGFAD.

## Materials and methods

The study methods received approval from the local institutional review board (IRB) at Jena University Hospital (registration number 2019-1505-MV), adhering to applicable guidelines and regulations. Given the retrospective nature of this investigation, the IRB waived the need for written informed consent.

### Dataset

**Fig. 1:**
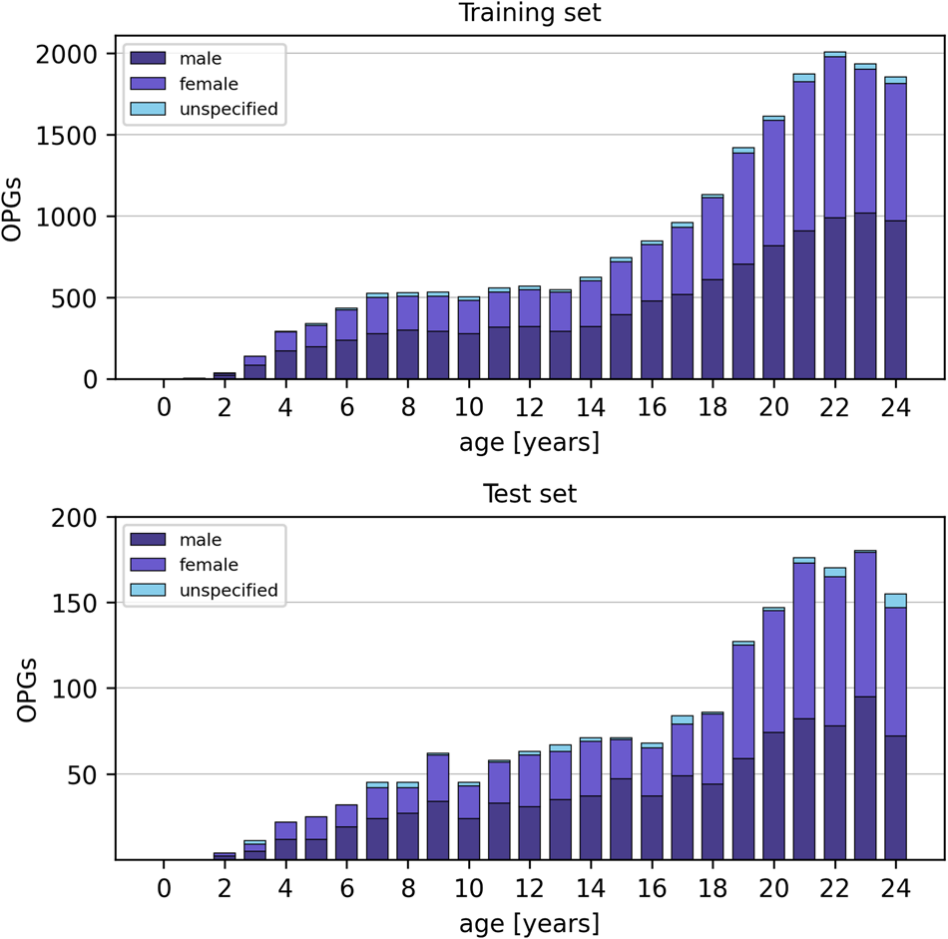
Distribution of age and sex in the training data (top) and test data (bottom). The sets consist of 20,000 and 1814 orthopantomograms (OPGs), respectively

For this study, 21,814 OPGs from 13,766 individuals aged 1 to under 25 years were obtained from the clinical image database, acquired between 2000 and 2023. Exclusion criteria included significant distortions, missing age information, and incomplete or unusable acquisitions. The data includes OPGs with resolutions ranging from 875 × 1688 pixels to 1552 × 3164 pixels. The most common resolutions are 875 × 1688 pixels (6967 images) and 1280 × 2440 pixels (2851 images) for 16-bit depth, as well as 1540 × 2948 pixels (2507 images), 1280 × 2440 pixels (2385 images), and 1552 × 2948 pixels (1690 images) for 8-bit depth. Devices used include Sirona with ORTHOPHOS-XG (9390 images), ORTHOPHOS-DS (3323 OPGs), and SIDEXIS (2129 OPGs), as well as ASEDV-HIPAX (6972 OPGs). Using the date of birth and acquisition date, the exact chronological age in days was calculated to improve the model’s accuracy. Afterwards, the data was randomly split into 1814 test samples and 20,000 training samples. Figure 1 shows an even age and sex distribution between the two groups. The mean age was 17.77 ± 5.72 years in the training- and 17.67 ± 5.63 years in the test data. Overall, the entire dataset consisted of 53% males, 45% females, and 3% of unspecified sex, with the majority of OPGs being of individuals aged 18 to under 25 years (59%).

All the OPGs were resized to 256 × 256 pixels, resulting in a distortion of the original rectangular shape into a square shape without cropping. To increase the diversity of the data, a data generator was used to randomly modify the existing OPGs in terms of slight zoom (0–20%), width (0–10%) and height shifts (0–20%), rotations (0–1°), and horizontal flips. For the process of learning, the training set was shuffled and divided into 80% training (16,000 OPGs) and 20% validation data (4000 OPGs).

### Custom CNN

The custom CNN has the same structure as stated in [18], comprising four blocks in total. Each block includes three convolutional layers followed by batch normalization, a max pooling, and a dropout layer. The learning rate was set to 0.0001, the dropout rate to 0.25, and the batch size to 64, as in the prior study. The number of filters, with a kernel size of 3 × 3, increases with each block, starting with 32 filters and doubling to 64, 128, and 256 filters, respectively. Following these four blocks, a fully connected layer with 1024 neurons is applied after flattening the output, along with batch normalization and a dropout layer. The CNN concludes with a fully connected layer containing a single neuron with linear activation to generate the final age output in days. The model contains a total of 69,076,289 parameters, occupying 263.5 MB. The implementation and training were performed using the Keras module in Python. The model was trained for 1000 epochs, with the training and validation data shuffled separately every 10 epochs. The best model was selected based on the lowest mean absolute error (MAE) and tested with 1814 OPGs. The results were statistically analyzed to investigate the influence of sex using a *U*-test with a significance level of 0.05, comparing the following categories: toddler age (1– < 3 years), preschool age (3– < 6 years), elementary school age (6– < 12 years), adolescence (12– < 18 years), and young adulthood (18–25 years). The final CNN architecture is accessible at: https://github.com/AG-TraFo/CNN-age-estimation.

### Transfer learning

For evaluation and comparison purposes, transfer learning was performed on a selection of well-established and widely used networks, including VGG-16, Inception-V3, DenseNet-201, ResNet-50, and EfficientNet-B4, utilizing pretrained weights from the ImageNet database. The same training, validation, and test OPGs used for the custom CNN were employed. For each base model, a flattening layer and two fully connected layers were added, with the first containing 1024 neurons and the second layer consisting of a single neuron, mirroring our architecture. The learning rate was set to 0.0001, and the batch size was 64. Early stopping was implemented with a patience of 10 epochs. Initially, only the added layers of the network were trained, with the ImageNet weights frozen. The best model obtained from this initial training phase underwent further training, during which all weights were allowed to be trainable. The best model for each transfer learning approach was selected based on the lowest MAE and tested with 1814 OPGs.

### Forensic age estimation

For this study, 15 forensic age assessments conducted by experienced experts certified by AGFAD were available. These assessments included all radiological procedures (OPGs, X-rays of the hand/wrist, thin-slice CT of the medial clavicular epiphyses) in addition to clinical examinations. In supplementary material, Fig. S1 presents a case example along with the referenced studies. Finally, the same OPGs were used to compare the results of forensic age estimation with those obtained from the custom CNN. It is important to note that for these cases, only the claimed age was available, not the proven chronological age.

## Results

**Fig. 2:**
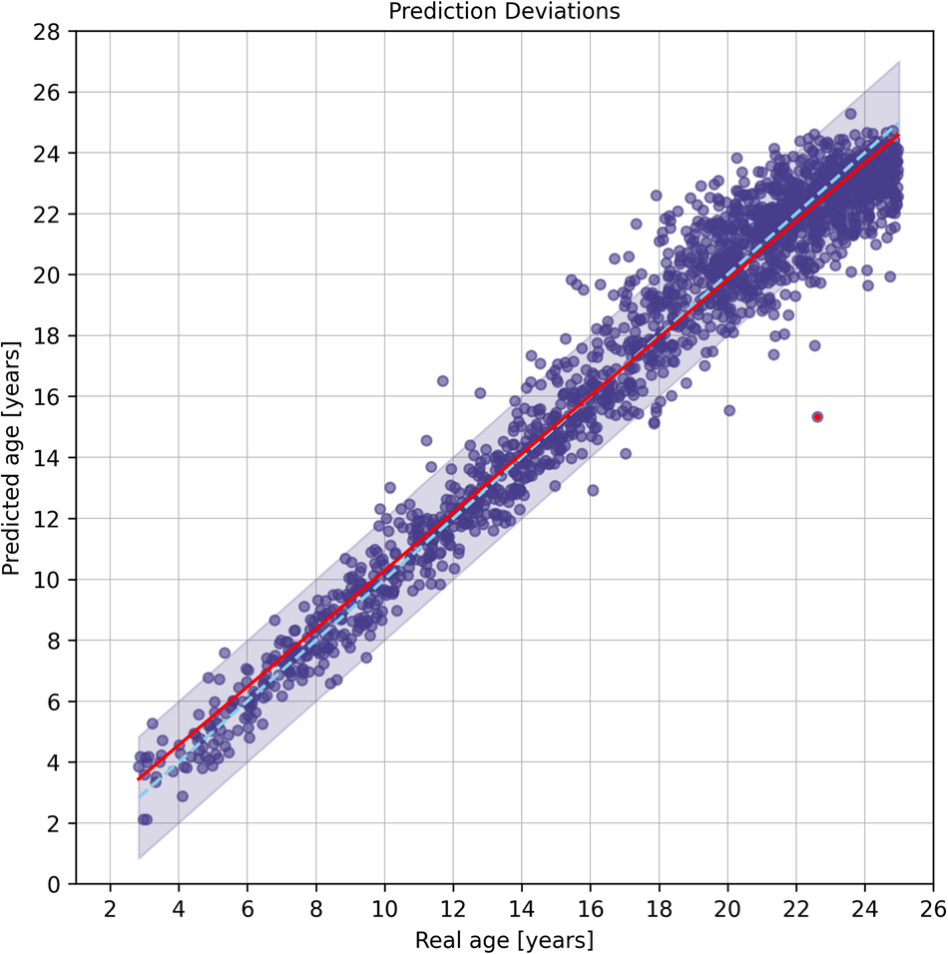
Results of the predicted age of 1814 independent test orthopantomograms (OPGs) compared to the actual chronological age. The red line represents the linear regression, the dashed blue line indicates the ideal case, and the shaded area illustrates a maximum deviation of 2 years. The red point demonstrates the image with the highest prediction deviation, which is displayed in Fig. 5f

**Fig. 3:**
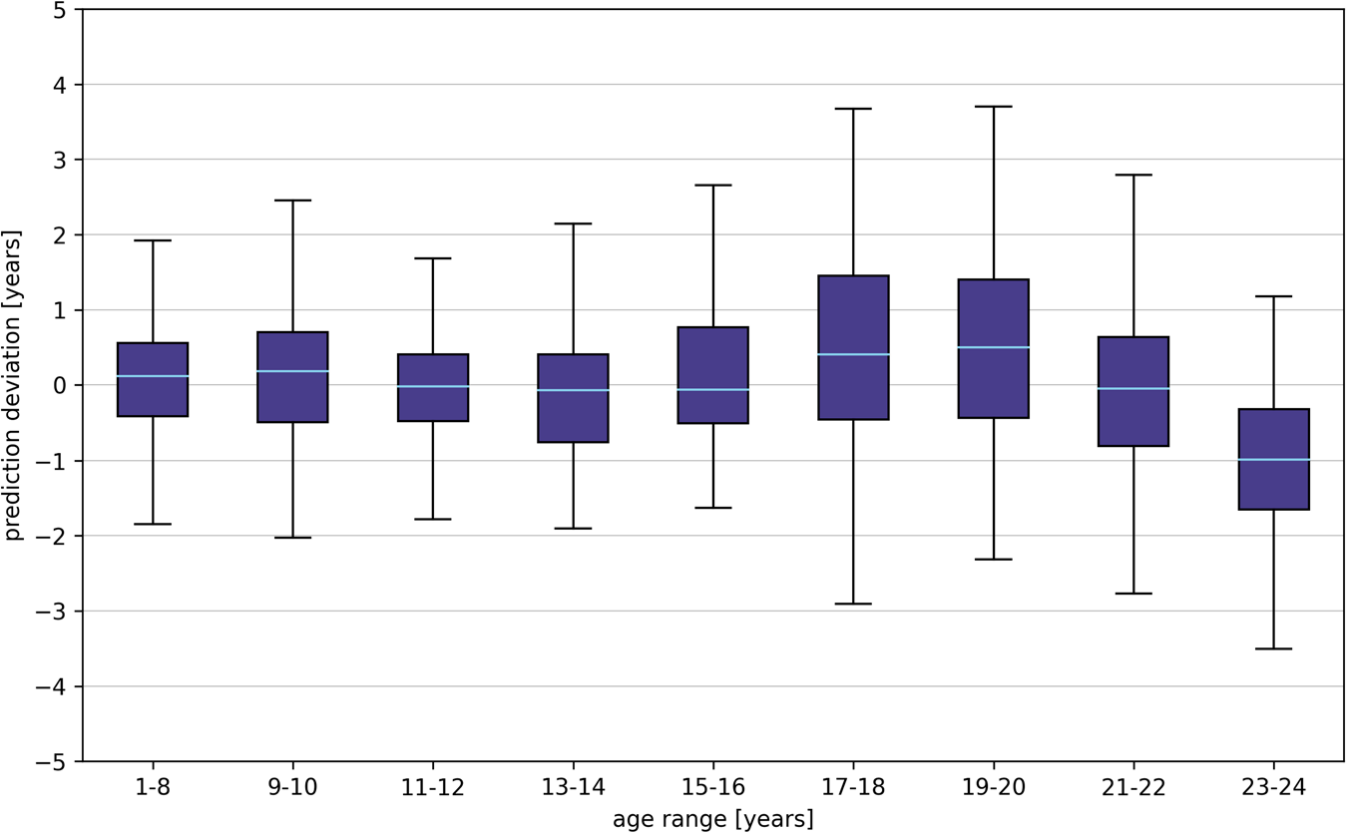
Boxplots showing the signed deviation between the prediction of the model and the real age of 1814 independent test data, divided into 9 different age groups. The whiskers indicate 1.5 times the interquartile range

The custom model demonstrates precision in age prediction, with an MAE of 0.93 ± 0.81 years and a mean-signed error (MSE) of −0.06 ± 1.23 years for all 1814 test OPGs. In Fig. 2, the data points along with prediction deviations are displayed, while Fig. 3 and Table 1 summarize the data as boxplots and numerical values, respectively. For 63% of the test data, the MAE is less than 1 year, and for 95% of the test OPGs, it is less than 2.50 years (refer to Table 2). In detail, in the age range of 1–8 years the MAE is lowest, at 0.57 ± 0.45 years. As age increases, the MAE rises to a maximum of 1.19 ± 0.98 years (age 17–18 years). The best median absolute error can be obtained in the age range of 11–12 years.

**Table 1:** Results from applying the stated model to age prediction on 1814 independent test orthopantomograms (OPGs), divided into age groups approximately the same size

Age (years)	OPGs	MED AE (years)	MAE (years)	SD (years)	MSE (years)	Sign. SD (years)
1–8	184	0.50	**0.57**	**0.45**	0.09	0.72
9–10	107	0.60	0.72	0.54	0.17	0.88
11–12	121	**0.45**	0.63	0.69	0.07	0.93
13–14	138	0.54	0.69	0.58	−0.02	0.90
15–16	139	0.60	0.87	0.84	0.21	1.19
17–18	170	1.00	1.19	0.98	0.51	1.46
19–20	274	0.92	1.11	0.81	0.48	1.28
21–22	346	0.74	0.94	0.83	−0.13	1.25
23–24	335	1.00	1.15	0.85	−1.05	0.98
Total	**1814**	**0.73**	**0.93**	**0.81**	**−0.06**	**1.23**

The median absolute error (MED AE) and mean absolute error (MAE) in context with the standard deviation (SD) represent the median and mean of the absolute deviations between actual and predicted ages. The mean-signed error (MSE) and the signed standard deviation (Sign. SD) indicate these differences by taking the sign of the deviation into account. The minimum values of MED AE, MAE, and SD are highlighted in bold, along with the complete metrics from the evaluation of the entire dataset

**Table 2:** Percentages and counts of orthopantomograms (OPGs) from the 1814 independent test cases, showing age predictions by the convolutional neural network (CNN) that fall within the specified maximum deviation from the actual age

Deviation (years)	Percentage	OPGs
< ± 0.5	36.5%	662
< ± 1.0	63.3%	1149
< ± 1.5	79.9%	1449
< ± 2.0	89.6%	1626
**< ± 2.5**	**95.2%**	**1727**
< ± 3.0	97.6%	1771
< ± 3.5	98.8%	1792
< ± 4.0	99.4%	1804
< ± 4.5	99.7%	1808
< ± 5.0	99.9%	1813

The highlighted row marks the deviation that first exceeds the significant threshold of 95%

**Fig. 4:**
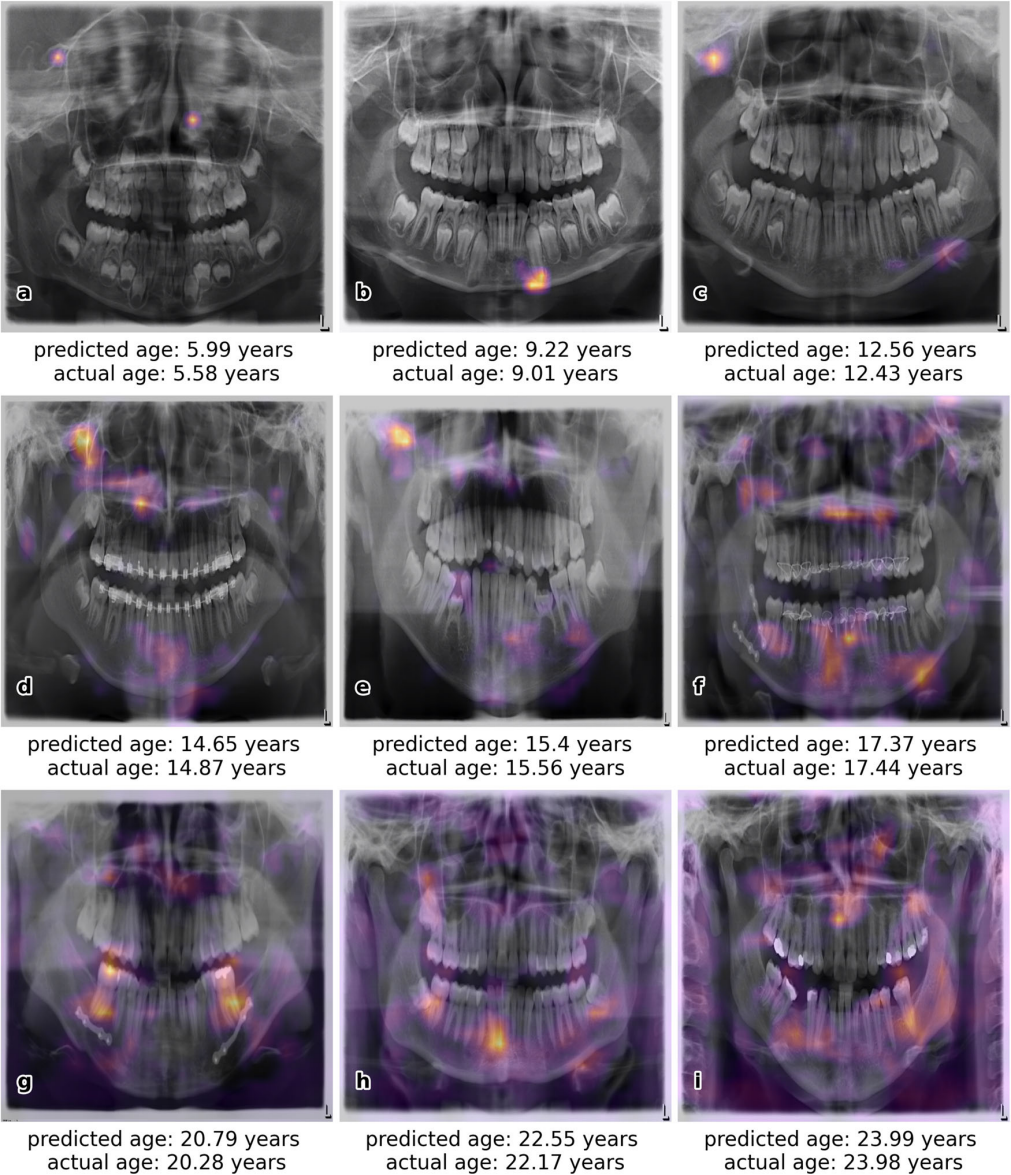
For each age group listed in Table 1, one example of a successful age prediction is provided ((**a**) 1–8 years, (**b**) 9–10 years, (**c**) 11–12 years, (**d**) 13–14 years, (**e**) 15–16 years, (**f**) 17–18 years, (**g**) 19–20 years, (**h**) 21–22 years, and (**i**) 23–24 years), including the respective orthopantomogram (OPG), the estimated age by the convolutional neural network (CNN), and the actual age. Additionally, Grad-CAM visualization is used to emphasize regions of the image, which are most essential for the specific age prediction

**Fig. 5:**
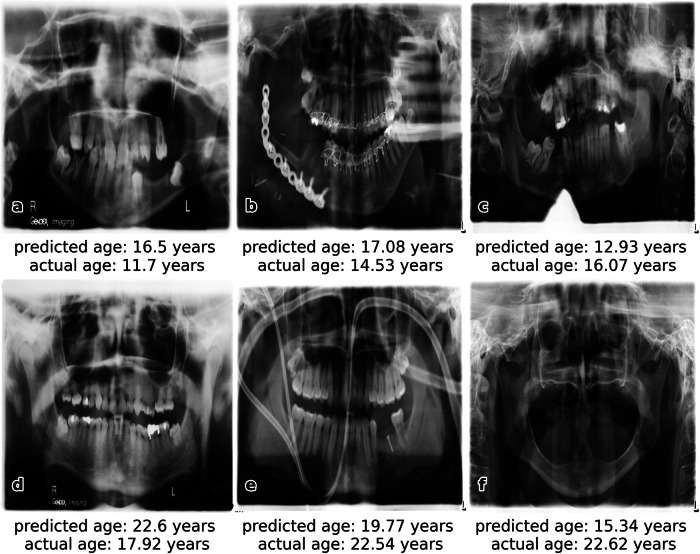
A selection of orthopantomograms (OPGs) of the test dataset with poorly predicted age. These OPGs deviate from the majority due to closed mouths with overlapping teeth (**a**, **c**), large implants (**b**), poor image quality, such as low contrast (**a**, **d**), the presence of medical equipment in the image (**e**), or the absence of teeth (**f**). The prediction of image (**f**) is the worst of all test images, presented as the red point in Fig. 2

Figure 4 illustrates successful examples of age estimation with Gradient-weighted Class Activation Mapping (Grad-CAM) visualization to highlight the CNN’s focus. Age estimation was also successful even in cases involving braces, implants, or dental work. In contrast, Fig. 5 displays examples of age estimations with larger deviations from the actual age. Reasons for poor performance in the estimation include OPGs that deviate from the majority, such as a closed mouth with overlapping teeth (a, c), large implants (b), poor image quality, e.g., low contrast (a, d), medical equipment in the image (e), or the absence of teeth (f).

The statistical analysis of the influence of sex on age estimation using the custom CNN revealed a significant difference in the age group 6–11 years (*p*-value = 0.005). All other age groups showed no significant differences between sexes, with *p*-values ranging from 0.066 to 0.667.

The determined best model (epoch index 912) achieved in the validation data a MAE of 0.99 years and a loss (mean squared error) of 1.81 years squared, and in the training set a MAE of 0.86 years and a loss of 1.18 years squared. The custom CNN demonstrates superior performance in age estimation accuracy compared to the transfer learning models VGG-16, Inception-V3, DenseNet-201, ResNet-50, and EfficientNet-B4 (refer to Table 3).

**Table 3:** Performance comparison of the presented CNN and transfer learning networks based on age prediction of 1814 independent test orthopantomograms (OPGs)

Model	MED AE (years)	MAE (years)	SD (years)	MSE (years)	Sign. SD (years)
custom CNN	0.73	0.93	0.81	−0.06	1.23
VGG-16	0.81	1.04	0.88	0.14	1.36
Inception-V3	0.85	1.07	0.91	−0.07	1.40
DenseNet-201	0.95	1.13	0.89	0.04	1.44
ResNet-50	1.11	1.36	1.08	−0.40	1.69
EfficientNet-B4	1.16	1.46	1.23	0.37	1.88

The metrics include the median absolute error (MED AE), mean absolute error (MAE) and standard deviation (SD), along with the mean-signed error (MSE) and signed standard deviation (Sign. SD)

**Fig. 6:**
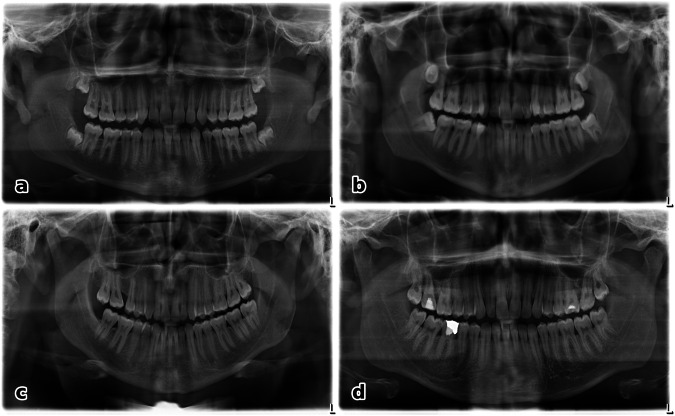
A selection of OPGs out of the 15 forensic age estimations. Images **a**, **b**, **c**, **d** correspond to individuals 1, 4, 8, 15 in Table 4

The results of forensic age diagnostics by experts in 15 cases (presented in Table 4) align closely with the CNNs age predictions, except for individual 1 (displayed in Fig. 6a). While the CNN estimates an age similar to the stated age, the forensic method indicates a greater age. In four cases (individuals 4, 7, 8, and 15), the age prediction of both methods suggests a different age than the claimed age.

**Table 4:** Results of 15 cases of forensic age estimation, conducted by experts by analyzing orthopantomograms (OPGs), hand X-rays, and clavicle computed tomography, leading to an estimation of the dental age, the minimum age, and the most likely age

		Forensic age diagnostics			CNN
Individual	Stated age (years)	dental age estimation (years)	Minimum age (years)	Most likely (years)	Prediction (years)
1	12.73	15.5	14	15.5	12.96
2	14.41	15.5	14	16	14.32
3	16.09	16	16	18	16.33
**4**	**17.32**	**19.5**	**19**	**22**	**21.67**
5	17.97	16.5	> 14	17	17.91
6	18.67	19.5	> 18	< 21	18.85
**7**	**18.67**	**21.5**	**> 21**	**> 21**	**21.26**
**8**	**18.68**	**21.5**	**> 21**	**> 21**	**23.04**
9	18.84	18.5	> 18	< 21	19.88
10	19.16	18.5	18	21	19.55
11	20.44	19.5	17.6	20.5	19.98
12	20.69	19.5	19	21	21.38
13	20.82	18.5	18	21	21.33
14	22.02	21.5	> 21	> 21	22.65
**15**	**22.85**	**18.5**	**> 18**	**19**	**20.49**

In comparison, the age prediction of the convolutional neural network (CNN) using the respective OPGs is presented. The highlighted rows marking significant deviations of the stated age from both age estimations. Only for individual 1 did the forensic result differ from the CNNs result

## Discussion

In this study, a custom CNN trained on 20,000 OPGs achieved high accuracy in age estimation, with MAE values below 1 year for 63% and below 2.50 years for 95% of 1814 tested OPGs, delivering results comparable to forensic experts. This innovative approach considers additional features beyond teeth and tooth roots, allowing estimation without preprocessing the OPG. Even at a resolution of 256 × 256 pixels, the CNN demonstrates excellent accuracy. The Grad-CAM visualization in Fig. 4 highlights key areas contributing to the model’s decision-making process. The heatmap is generated by using the gradients of the target class (the age), flowing into the final convolutional layer [20]. The CNN utilizes both dental and non-dental features from the entire head region, such as the jaw, periodontal membrane, paranasal sinus, and maxillary sinus, enhancing the reliability of age estimation. However, difficulties arise when OPGs significantly deviate from the norm, such as in cases of closed mouths with overlapping teeth, large implants, poor image quality, visible medical equipment, or absence of teeth. The only OPG in the test data that lacked teeth and dental prostheses (Fig. 5f) produced the poorest age estimation results. A contributing factor to this is the occurrence of only five fully edentulous OPGs in the training data, along with 16 OPGs that were edentulous in either the upper or lower jaw. A key advantage in real forensic cases is that they typically involve healthy individuals, resulting in high-quality OPG images not affected by severe accidents or surgeries. Finally, this study relies on accurate age information in the Digital Imaging and Communications in Medicine (DICOM) header, which may be affected by transmission errors or misreporting.

This study utilizes one of the largest datasets of OPGs in the literature [6–17], featuring significant diversity in resolution, bit depth, and devices used. In the previous study [18], it was demonstrated that the accuracy of a CNN increases with more OPGs in the training set for the same model. The dataset includes both healthy and diseased individuals, as well as those with and without (dental) implants. This diversity enhances the generalizability of the results for real-world applications. Furthermore, the model and its weights are available for download (https://github.com/AG-TraFo/CNN-age-estimation), enabling further training on custom datasets (transfer learning) or direct application. In Table 5, a selection of comparable studies [6–17] is summarized, although it is not exhaustive due to the numerous studies available. Han et al [11] achieved a MAE of 0.83 years with the application of EfficientNet for 1017 tested OPGs aged 5 to 24 years, with measurement uncertainty increasing with age. Comparing the MAE between different studies is generally challenging. Considering the number of OPGs, the MAE for the following three groups between our study and Han et al [11] was 0.63/0.47 years (6– < 12 years), 0.82/1.00 years (12– < 18 years), and 1.08/1.11 years (18–25 years), indicating that our study demonstrates higher accuracy in the relevant age range. Additionally, Han et al [11] excluded OPGs based on criteria such as systemic diseases, prior dental treatments, dental trauma, anomalies, missing lower teeth, jawbone pathologies, and poor-quality radiographs, which may have limited the generalizability of their results. In contrast, Vila-Blanco et al [8] also included OPGs with poor quality and conditioning dental characteristics but reported a higher MAE of 1.17 ± 1.11 years. Other models, such as those by Aliyev et al [12], which achieved an MAE of 0.64 years for female individuals, performed better but worked with smaller datasets and more specific age groups, potentially limiting their generalizability. This also applies to Wang et al [13], Büyükçakır et al [16], and Shi et al [17], who utilized relatively smaller datasets. Other studies [6, 7, 9, 10, 14, 15] used classification into age groups, but the incorrect assignment of an age group can lead to significantly larger errors due to the considerable distance between groups. In contrast, regression, as demonstrated in this study, typically remains closer to the actual value, even if the estimate is not perfect. Moreover, it has been demonstrated that an OPG can be used directly with a CNN, eliminating the need for additional segmentations of individual teeth [6, 9, 12, 15, 17] to achieve high accuracy, thus simplifying the process.

**Table 5:** Overview of literature on forensic age estimation using convolutional neural networks (CNNs) and orthopantomograms (OPGs)

Publication	Regression/Classification	Model	Data	Age (years)	Method	Performance
Kahaki et al, 2019 [6]	Classification into 5 age groups at a 3- or 4-year interval	Deep CNN	812 OPGs, train: 356 OPGs, test: 456 OPGs	1–17	Segmentation of the first-to-third molar using global fuzzy segmentation, followed by local feature extraction with a projection-based feature transform and a deep CNN for age classification.	The average classification rate was 81.83%. The rate in the specific age group 1–4, 5–7, 8–10, 11–13 and 14–17 was 80.05%, 77.15%, 80.15%, 85.37% and 86.45%, respectively.
Mualla et al, 2020 [7]	Classification into 8 age groups at a 10-year interval	AlexNet/ResNet-101	1429 OPGs resized to 277 × 277 /224 × 224 pixels, 10-fold cross-validation with train: 1286 OPGs, test: 143 OPGs	0– > 70	Feature extraction with AlexNet/ResNet-101 and classification with various classification models.	The K-NN classifier performs best with an accuracy of 98.8% for both AlexNet and ResNet.
Vila-Blanco et al, 2020 [8]	Regression	DentalAgeNet (DANet), DentalAgeandSexNet (DASNet)	2289 OPGs, resized to 256 × 128 pixels, 8-fold cross-validation with train: 1465 OPGs, validation: 366 OPGs, test: 458 OPGs	4.5–89.2	Development of two CNN architectures. One estimates only the age, the other also has links to an additional CNN, which classifies the sex, to improve the age estimation.	The DASNet performed best, with a MAE of 1.17 ± 1.11 years for the dataset ${S}_{ > 25}$, with all subjects under 25 years of age.
Kim et al, 2021 [9]	Classification into age groups (0–19, 20–29, 30–39, 40–49 and over 50)	ResNet-152	OPG-patches of the first four molars from 1586 individuals, resized to 151 × 112 pixels, train: 1078 OPGs, validation: 190 OPGs, test: 318 OPGs	0– > 60	Tooth-wise age group estimation with the four molars separately and assignment to the age group with the highest number of votes.	For the combined patient-wise classification into the 5 age groups, an accuracy of 90.37 ± 0.93% was achieved.
Guo et al, 2021 [10]	Classification (age thresholds 14, 16, 18)	CNN models based on EfficientNet/SE-ResNet-101	10,257 OPGs, train: 80% validation: 10% test: 10%	5–24	Development of several CNNs, that indicate, whether the individual is over or under the age 14, 16 and 18, respectively, and comparing the results to manually determined classification.	The CNN models perform better with accuracies of 95.9%, 95.4% and 92.3% compared to 92.5%, 91.3% and 91.8% of the manual method for the age thresholds 14, 16 and 18, respectively.
Han et al, 2022 [11]	Regression	ADSE (based on ResNet-101) and ADAE (based on EfficientNet)	10,257 OPGs, train: 8325 OPGs validation: 915 OPGs test: 1017 OPGs	5–24	Comparison of age estimation via a linear regression model based on the sex and estimated stages of permanent teeth by a CNN (ADSE) and a fully automated age estimation model with feature extraction without human interference (ADAE).	The ADSE model achieved an MAE of 0.17 stages in stage estimation and 1.63 years in age estimation. The ADAE model outperformed ADSE with an MAE of 0.83 years.
Aliyev et al, 2022 [12]	Classification and Regression	CNN for classification, regression with LightGBM	475 OPGs, segmented teeth had a resolution of 224 × 224 pixels, 10-fold cross-validation with train: 304 OPGs, validation: 76 OPGs, test: 95 OPGs	6–13.8	Segmentation of the images to classify the developmental stages of the hidden teeth at the left mandibula with a CNN and regression for age estimation using the stages.	The age estimation error for females was on average 0.64 years with a min of 0.025 years and a max of 2.25 years. For males, the mean error was 0.6 years with a min of 0.0 years and a max of 2.75 years.
Wang et al, 2022 [13]	Regression	DENSEN	1903 OPGs, resized to 447 × 447 pixels, train: 1647 OPGs, test: 256 OPGs	3–85	Development and application of the DENSEN network, derived from SSR-Net.	The MAEs in the age groups 3–11, 12–18 and 19–25 years are 0.6885, 0.7615 and 1.3502, respectively.
Baydogan et al, 2023 [14]	Classification into 3 classes (2–6, 6–13, 13–21 years)	Age-Net (EfficientNetB0-SVM hybrid model)	933 OPGs, resized to 224 × 224 pixels, 5-fold cross-validation with train: 746 OPGs test: 187 OPGs	2–21	Extraction of feature vectors from the images with various CNNs and detection of the age with varying algorithms.	The combination of the EfficientNetB0 and the Support Vector Machine (SVM) performed best, with an accuracy of 84.6%.
Hemalatha et al, 2023 [15]	Classification	Deep CNN	100 OPGs sized 275 × 158 pixels, train: 60 OPGs, test: 40 OPGs	4–18	Segmentation of the images with Fuzzy C-Means Clustering, followed by optimal feature score selection with Ant Lion Optimization and age classification using a deep CNN.	The accuracy of the age classification was 91%.
Büyükçakır et al, 2024 [16]	Regression	EfficientNet-B4, DenseNet-201, MobileNet V3, VGG-16	4367 OPGs resized to 450 × 250 pixels, 5-fold cross-validation with train: 3896 OPGs test: 454 OPGs	0–25	Exploration of the impact of parameters on a CNN for age estimation through variations in hyperparameters, model complexity, batch size, and sample quantity.	EfficientNet-B4 performed best, trained with the complete dataset and a batch size of 160. The MAE was 0.562 years.
Shi et al, 2024 [17]	Classification and Regression	SOS-Net (based on YOLOv3 and ResNet-50)	673 OPGs, train: 471 OPGs, validation: 68 OPGs, test: 134 OPGs	3–14	Detection and numbering of the permanent teeth with a model based on YOLOv3, identification of the maturity of the teeth with a novel SOS-ResNet-50, and statistical meta-analysis for age estimation.	Overall, the method achieved an MAE of 0.72 years by using the full dentition.
Prior study Heinrich, 2024 [18]	Regression	Deep CNN	60,779 OPGs resized to 256 × 256 pixels, train: 40,000 OPGs, validation: 10,000 OPGs, test: 10,779 OPGs	2–89	Application of the stated CNN with custom architecture across the entire age range from 2 to 89 years.	An overall MAE of 2.76 ± 2.67 years for postmortem OPGs and 3.26 ± 3.06 years for antemortem OPGs was achieved. The MAEs for the age groups 2–9, 10–19, and 20–29 years are 1.48, 1.44, and 1.99 years, respectively.
This study	Regression	Deep CNN	21,814 OPGs resized to 256 × 256 pixels, train: 16,000 OPGs, validation: 4000 OPGs, test: 1814 OPGs	1–24	Application of the stated CNN with custom architecture.	An overall MAE of 0.93 ± 0.81 years was achieved, with 63% within a 1-year deviation, and 95% within a 2.5-year deviation.

The choice of hyperparameters is vital for CNN performance; however, GPU architecture and memory limitations can influence this choice [16]. Key factors like batch size, learning rate, and dropout rates are significant for the model’s learning ability and generalization. Larger batch sizes can accelerate convergence but may require more powerful hardware, particularly more memory, to process larger datasets simultaneously. An optimal learning rate is crucial; a rate that is too high can cause overshooting, while a rate that is too low prolongs training. Dropout rates help prevent overfitting and promote robust feature learning. Careful tuning of these hyperparameters can improve model accuracy. The prior study [18] systematically evaluated various hyperparameters, leading to the selection of the optimal settings used in this study. The analysis demonstrates that our custom CNN effectively estimates age across a broad range, from 1 to 25 years, independent of sex. The significant difference observed in the 6–11 age group suggests that while sex may influence age estimation in certain developmental stages, CNN maintains a robust performance overall. This highlights its capability to generalize across diverse populations. The lack of significant differences in other age groups reinforces the model’s adaptability, indicating that it can accurately assess age without being biased by sex.

Several comparative studies have demonstrated the superiority of CNNs over traditional methods like Demirjian’s [10, 21]. Manual age estimation techniques, such as those by Demirjian, Willems, Cameriere, Nolla, Smith, Haavikko, and Chaillet, primarily developed for children and adolescents, determine chronological age by assessing dental features observed in X-rays [22, 23]. However, these methods are time consuming and are prone to subjective judgment and often exhibit a wide margin of error, as highlighted in a review [23] showing contrasting results for the same population. Research suggests average error rates of around ± 1–2 years for children in general [24], due to different rates of dental development in children. Furthermore, age estimation in younger individuals is more precise due to the rapidly changing dentition during developmental stages [25].

This study has several limitations. First, the datasets were obtained from a single hospital, making them region-specific and potentially limiting the generalizability of the findings. Another limitation is that OPGs are only one component of forensic age estimation, alongside other methods like clavicle analysis. Additionally, the study lacked intervals for classifying minors and adults and did not establish minimum age thresholds with high certainty, focusing solely on point age predictions. These intervals are crucial for practical forensic applications. Future research could combine various CNNs for classification (e.g., minors vs. adults; minimum ages > 14, > 18, > 21 years). If multiple CNNs yield consistent results, this could enable the introduction of intervals with higher certainty. Furthermore, the application of a CNN functions as a black box, making it difficult to trace how age estimations are made or assess their reliability. Finally, further investigation is needed to explore the relationship between chronological and biological age in CNN-based estimations, as the observed error margin of less than 1 year may arise from discrepancies between actual and physiological age, influenced by genetics, lifestyle, and environmental factors.

In conclusion, a custom CNN for age estimation has been developed, proving high accuracy after training on the largest OPG dataset to date, managing various factors such as resolution, bit depth, devices, health status, and dental implants. Notably, its performance is mostly sex-independent. By making the model and weights publicly available, we aim to support further research and applications. This also allows transfer learning with custom data, benefiting from robust pre-learned features. Furthermore, the application of this CNN does not require additional imaging, making it an easily implementable new tool for AGFAD-certified experts, thereby potentially enhancing the safety and reliability of the three-step approach for legal cases in the future.

## Supplementary information

ELECTRONIC SUPPLEMENTARY MATERIAL

## Abbreviations

CNN: Convolutional neural network
Grad-CAM: Gradient-weighted Class Activation Mapping
MAE: Mean absolute error
MSE: Mean-signed error
OPG: Orthopantomogram
