# Supplementary material for: Deep learning for forensic age estimation using orthopantomograms in children, adolescents, and young adults
Source: Eur Radiol. 2025 Jan 25;35(7):4191–202. doi: 10.1007/s00330-025-11373-y (PMC12165891; doi:10.1007/s00330-025-11373-y)
Supplement: Supplementary file 1 — ELECTRONIC SUPPLEMENTARY MATERIAL [file 330_2025_11373_MOESM1_ESM.pdf]

Deep Learning for forensic age estimation using orthopantomograms in children, adolescents, and young adults

ELECTRONIC SUPPLEMENTARY MATERIAL

Eur Radiol (2025) Koch RM, Mentzel HJ, Heinrich A.

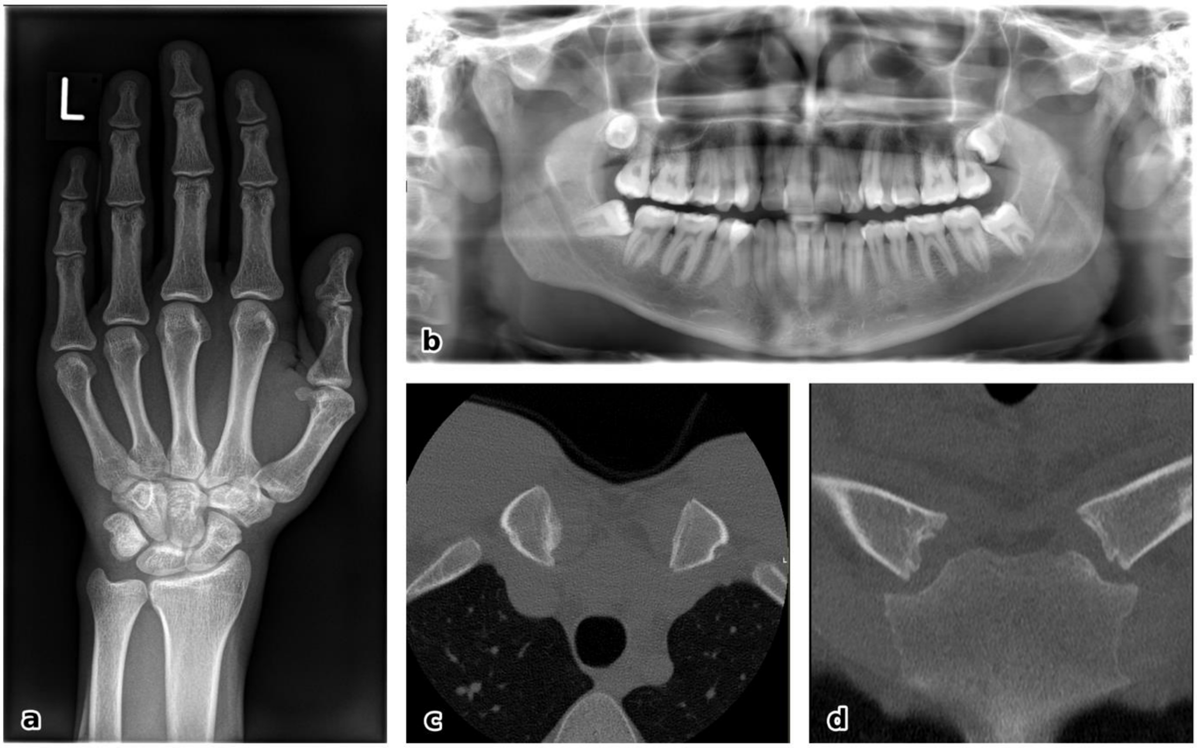

Figure S1 This figure shows an example of age estimation using X-ray of the left hand (a), OPG (b), and CT of the medial clavicular epiphyses in axial (c) and coronal (d) planes. The age assessment was conducted as follows: a) Mineralization is without abnormalities. All epiphyseal plates are closed, with a faint scar visible on the radius and the first metacarpal bone. Based on Greulich & Pyle [1], the skeletal age of this male hand is 19 years. The absolute minimum age for complete hand skeletal ossification in males is 16.1 years [2]. b) According to the FDI dental chart, teeth 18 and 28 are not clearly assessable. Teeth 38 and 48 are at mineralization stage G [3], with open root apices and fully developed crowns. The mean chronological age for stage G of teeth 38 and 48 is 21.3 years [4], with a standard deviation of 2 years for tooth 38 and 2.1 years for tooth 48. c-d) CT reconstruction with 0.625 mm slice thickness shows a subtle fishmouth configuration on the right medial clavicle end, making it impossible to assign a specific ossification stage. On the left, the ossification center is clearly demarcated, with partial fusion between the meta- and epiphysis. The cranial part of the axial surface is already fused, visible as a sclerosis band. In the coronal reconstruction, approximately two-thirds of the surface is fused, corresponding to stage 3c [5]. The mean chronological age for males at stage 3c is 23.6 years, with a standard deviation of 2.6 years [6]. The absolute minimum age for this stage is 19 years. In summary, the absolute minimum age is 19 years, with a probable age of around 22 years based on the preponderance of evidence.

References

1 Greulich WW, Pyle SI (1959) Radiographic Atlas of Skeletal Development of the Hand and Wrist. Stanford University Press

2 Tisè M, Mazzarini L, Fabrizzi G, Ferrante L, Giorgetti R, Tagliabracci A (2011) Applicability of Greulich and Pyle method for age assessment in forensic practice on an Italian sample. International journal of legal medicine 125:411-416

3 Demirjian A, Goldstein H, Tanner JM (1973) A new system of dental age assessment. Human biology:211-227

4 Olze A, Taniguchi M, Schmeling A et al (2003) Comparative study on the chronology of third molar mineralization in a Japanese and a German population. Legal Medicine 5:S256-S260

5 Kellinghaus M, Schulz R, Vieth V, Schmidt S, Pfeiffer H, Schmeling A (2010) Enhanced possibilities to make statements on the ossification status of the medial clavicular epiphysis using an amplified staging scheme in evaluating thin-slice CT scans. International journal of legal medicine 124:321-325

6 Wittschieber D, Schulz R, Vieth V et al (2014) The value of sub-stages and thin slices for the assessment of the medial clavicular epiphysis: a prospective multi-center CT study. Forensic Science, Medicine, and Pathology 10:163-169
